# Aktuelle Graduierung des Prostatakarzinoms

**DOI:** 10.1007/s00292-025-01446-6

**Published:** 2025-07-02

**Authors:** Yosamin Gonzalez Belisario, Geert J. L. H. van Leenders

**Affiliations:** 1 Abteilung für Pathologie, Erasmus MC Cancer Institute, University Medical Centre, Rotterdam, Niederlande; 2 Department of Pathology, Raum Be-222, Erasmus MC Cancer Institute, University Medical Centre, 2040, 3000 Rotterdam, CA Niederlande

**Keywords:** Wachstumsmuster, Kribriformes Muster, Intraduktales Karzinom, Klassifizierungssystem, Gleason-Score, Growth pattern, Cribriform pattern, Intraductal carcinoma, Classification system, Gleason score

## Abstract

Das Gleason-Graduierungssystem bildet die Basis der Risikostratifizierung und der klinischen Entscheidungsfindung bei Prostatakrebspatienten. In den letzten zehn Jahren haben neue Erkenntnisse über die quantitative Einstufung und die Bedeutung bestimmter Wachstumsmuster die histopathologische Charakterisierung von Tumoren weiter optimiert. In dieser Übersichtsarbeit werden die neuesten Empfehlungen für die Graduierung vorgestellt und aktuelle Forschungsrichtungen erörtert.

## Abstract (en)

The Gleason grading system is the cornerstone of risk stratification and clinical decision-making in prostate cancer patients. In the last decade, new insights on quantitative grading and the importance of particular growth patterns have further optimized pathological tumor grading. This review provides an update on the latest grading recommendations and discusses contemporary research directions.

Prostatakrebs (PCa) ist der am häufigsten diagnostizierte maligne Tumor und die fünfthäufigste Ursache für krebsbedingte Sterblichkeit bei europäischen Männern. Das Gleason-Klassifizierungssystem ist seit mehr als einem halben Jahrhundert für die Risikostratifizierung und die klinische Entscheidungsfindung bei PCa-Patienten von wesentlicher Bedeutung. Im Laufe der Jahre wurden mehrere Modifikationen des Gleason-Scores (GS) eingeführt. Diese Übersicht gibt einen Überblick über die neuesten Empfehlungen zur Einstufung und zeigt zukünftige Forschungsrichtungen auf.

## Die Gleason-Score-Klassifikation und ihre Veränderungen

Im Jahr 1966 führte die Veteran’s Administration Cooperative Urological Research Group (VACURG)-Studien zum Vergleich verschiedener Behandlungsmodalitäten für das PCa durch, dies schloss eine Bewertung einer breiten Palette potenziell prognostischer Parameter ein. Eine der wichtigsten Komponenten war die pathologische Kategorisierung der Tumoren, die von Dr. Gleason, dem für die Studie zuständigen Pathologen, akribisch durchgeführt wurde [1]. Es zeigte sich, dass die Wachstumsmuster des Tumors für die Prognose wichtiger waren als die Mitosefrequenz oder zytologische Merkmale. Basierend auf ähnlicher prognostischer Bedeutung wurden spezifische Wachstumsmuster letztlich zu 5 Gleason-Mustern („gleason pattern“, GP) zusammengefasst, welche von gut differenzierten, dicht gepackten Drüsen bis hin zu undifferenzierten Karzinomen mit minimaler oder gar keiner Drüsenbildung reichen. Da das PCa als heterogener Tumor häufig aus mehr als einem GP besteht, entwickelte Dr. Gleason ein Klassifizierungssystem, in dem das vorherrschende (primäre) und das sekundäre Muster zu einem GS von 2–10 zusammengefasst wurde.

Die histopathologische Diagnostik zu Gleasons Zeiten unterschied sich erheblich von der heutigen Praxis im 21. Jahrhundert. PCa wurden häufig entweder in einem späten Stadium aufgrund klinischer Symptome diagnostiziert oder zufällig bei transurethralen Prostataresektionen (TURP) entdeckt, die zur Behandlung einer obstruktiven Symptomatik durchgeführt wurden. Auch die Messung des prostataspezifischen Antigens (PSA) im Serum und die Immunhistochemie waren damals nicht Teil der Untersuchung. Mutmaßlich würde man einige der gut differenzierten GS < 4-Tumoren, die in den 1960er- bis 1980er-Jahren bei der TURP diagnostiziert wurden, heute als Adenose/atypische adenomatöse Hyperplasie einordnen, was allerdings oft eine (damals nicht verfügbare) Immunhistochemie erfordern würde.

Im Jahr 2005 hielt die Internationale Gesellschaft für Urologische Pathologie (ISUP) eine Konsenskonferenz ab, auf der die Empfehlungen für die Gleason-Graduierung auf der Grundlage der neuesten wissenschaftlichen Erkenntnisse und der veränderten klinischen Praxis erheblich aktualisiert wurden, was zu einem modifizierten GS führte [2]. So wurde beispielsweise vereinbart, dass ein GS < 5 in Nadelbiopsien „selten, wenn überhaupt“ vergeben werden sollte. Darüber hinaus sollten die meisten kribriformen Muster als GP4 klassifiziert werden, wobei nur wenige Ausnahmen von kleinen kribriformen Drüsen noch mit GP3 vereinbar sind [2].

**Abb. 1:**
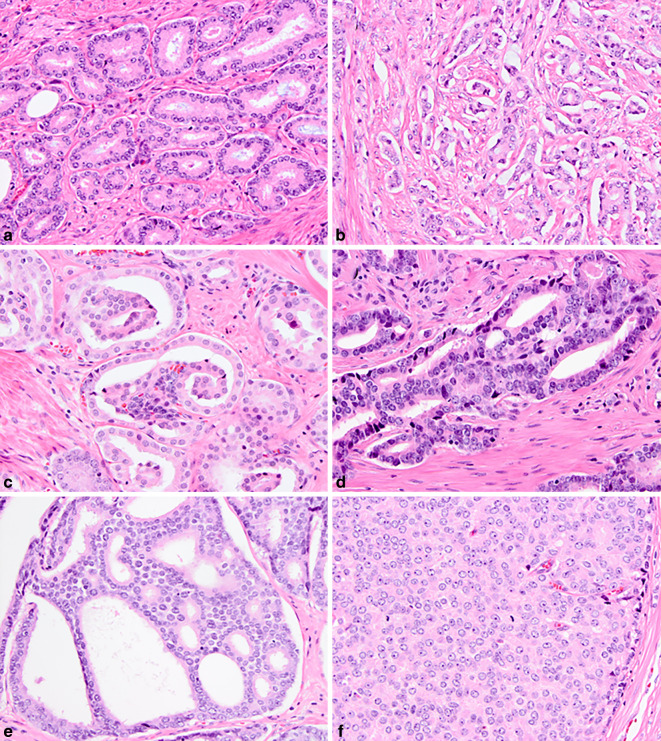
Übersicht der Gleason-Muster („Gleason pattern“, GP) des Prostatakarzinoms. **a** GP3 – gut geformte Tubuli. **b**–**e** GP4 – schlecht-geformte Tubuli (**b**), glomeruloide (**c**), fusionierte Drüsen (**d**) und kribriformes Wachstum (**e**). **f** GP5 – solide Tumorzellverbände (Hämatoxilin-Eosin [HE]-Färbung, 200×)

Auf der nachfolgenden ISUP-Konsenskonferenz in Chicago 2014 wurden weitere geringfügige Änderungen eingeführt, beispielsweise, dass auch kleine kribriforme Muster und glomeruloide Drüsen dem Spektrum dieses GP zugeordnet werden sollten [3]. Darüber hinaus wurde ein Vorschlag von Dr. Epstein zur Gruppierung des GS in ISUP-Graduierungsgruppen (GG) in Verbindung mit dem GS angenommen: GS ≤ 6 (GG1), GS3 + 4 = 7 (GG2), GS4 + 3 = 7 (GG3), GS8 (GG4) und GS9–10 (GG5; [4]). Die klinischen Vorteile der neuen GG waren die klare Unterscheidung zwischen GS3 + 4 und 4 + 3 sowie eine Verbesserung der Patientenkommunikation durch die Zuordnung von GG1 zu den Tumoren mit dem geringsten Risiko anstelle von GS6 (innerhalb eines Spektrums von 2–10). Seit der ISUP-Konsenskonferenz 2014 umfassen die GG die folgenden Wachstumsmuster: GG1 ist durch einzelne, umschriebene, reife Tumordrüsen gekennzeichnet; GG2 ist eine Mischung aus diesen gut ausgebildeten Drüsen mit einer sekundären Komponente von schlecht ausgebildeten, fusionierten, glomeruloiden oder kribriformen GP4-Drüsen, während in GG3 GP4 das vorherrschende Muster ist. GG4 stellt die heterogenste Gruppe dar, die nicht nur GS4 + 4 = 8, 3 + 5 = 8 und 5 + 3 = 8 umfasst, sondern auch eine große Vielfalt an relativen GP-Anteilen und individuellen Wachstumsmustern aufweist. GG5 umfasst einzelne Zellen, Stränge, Nester, solide Formationen oder Komedonekrosen, die alle mit GP5 gekennzeichnet sind, allein oder in Kombination mit GP4 (Abb. 1; [3]).

**Abb. 2:**
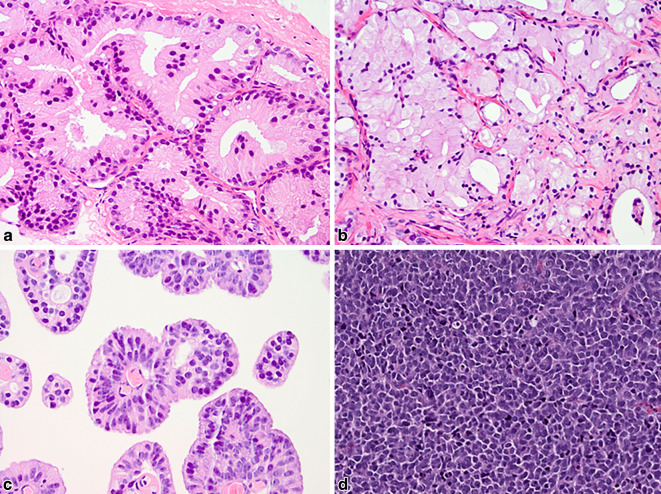
Überblick über ungewöhnliche Muster des Prostatakarzinoms (**a**,**b**) und Subtypen (**c**,**d**): **a** Pseudohyperplastisches Karzinom, dicht gepackte atypischen Drüsen mit zarten papillären Einfaltungen (GP3). **b** Schaumzelliges Karzinom mit reichlich schaumigem Zytoplasma und exzentrisch lokalisierten kleinen Kernen; das Gleason-Muster (GP) orientiert sich an der Architektur, jedes GP möglich. **c** Duktales Adenokarzinom mit papillären Strukturen, bedeckt von schlankem Zylinderepithel mit elongierten Kernen (GP4; GP5, wenn Nekrosen vorliegen). **d** Kleinzellig Neuroendokrines Karzinom, welches nicht graduiert wird (Hämatoxilin-Eosin [HE]-Färbung, 200×)

Es gibt einige ungewöhnliche histologische Muster des azinären Adenokarzinoms, darunter atrophe oder schaumzellige Drüsen, pseudohyperplastische, muzinöse und der prostatischen intraepithelialen Neoplasie (PIN) ähnelnde, PIN-artige Karzinome, die aber alle nach ihrer Histoarchitektur graduiert werden sollten, wobei es sich meist um das GP3 handelt (Abb. 2a,b). Neben diesen archetypischen azinären Karzinomwachstumsmustern werden die seltenen papillären azinären Adenokarzinome und duktale Karzinome (ohne Nekrosen) dem GP4 zugeordnet (Abb. 2c; [5]). Kleinzellige neuroendokrine oder sarkomatoide Karzinome, welche überwiegend in späten Erkrankungsstadien auftreten, sind per definitionem „high grade“ und werden nicht nach Gleason graduiert (Abb. 2d).

## Quantitative Bewertung der Gleason-Muster

Die quantitative Einschätzung der GP ist ein Eckpfeiler des GS. Dies wird am besten im GS7 veranschaulicht, wo die Anteile der GP3 und 4 bestimmen, ob ein Tumor der GG2 (3 + 4) oder GG3 (4 + 3) zugeordnet wird. Es gibt jedoch auch Bereiche mit Unklarheiten, z. B. bezüglich der Berücksichtigung des minimalen GP-Volumens, der Koexistenz von GP3, 4 und 5 und der Einstufungsregeln für Biopsie- und radikale Prostatektomieproben (RPE).

Auf der letzten ISUP-Konsenskonferenz 2019 wurden die folgenden Empfehlungen zu diesen Fragen formuliert [6]: Wenn GP3 < 5 % des Tumorvolumens ausmacht, wird es nicht in den GS/GG von Biopsie- und RP-Proben einbezogen. Bei Biopsien mit einem geringen Prozentsatz an höhergradigen Mustern wird dieser immer als sekundärer Grad des GS berücksichtigt, während bei RP-Proben ein Schwellenwert von ≥ 5 % überschritten werden sollte. Wenn das hochgradige Muster im Ektomiepräparat < 5 % ausmacht, sollte es im GS bzw. der GG nicht berücksichtigt werden, aber sein Vorhandensein sollte als geringfügiges oder tertiäres Muster angegeben werden. Bei einem einzelnen Knoten, der zu 60 % das Muster 3, zu 25 % das Muster 4 und zu 15 % das Muster 5 aufweist, würde das endgültige GS sowohl in der Biopsie als auch im Ektomiepräparat 3 + 5 = 8 betragen [7]. Ein Tumor mit 60 % Muster 3, 38 % Muster 4 und 2 % Muster 5 würde bei der Biopsie als GS3 + 5 = 8 (GG4) eingestuft werden, in der RPE jedoch als 3 + 4 = 7 (GG2) mit Neben‑/Tertiärkomponente Muster 5 (2 %).

Während die GP-Anteile und Grenzwerte die Einstufung in GS und GG bestimmen, lässt sich durch eine weitergehende Quantifizierung der GP-Menges eine zusätzlichen Risikostratifizierung erreichen. In einer großen Studie basierend auf einer Kohorte von 12.000 Männern nach Prostatektomie zeigten Sauter et al. [8], dass das Risiko eines biochemischen Rezidivs mit zunehmendem GP4-Anteil graduell ansteigt. Auf der Grundlage dieser und anderer Studien empfehlen die Weltgesundheitsorganisation (WHO), die ISUP und die International Collaboration of Cancer Reporting (ICCR) die Angabe des prozentualen GP4-Anteils bei GS7-PCa [5, 6, 9, 10]. Abgesehen von der prognostischen Information wird der prozentuale Anteil von GP4 in GS3 + 4 = 7 (GG2) als Auswahlkriterium für einige aktive Überwachungsprotokolle verwendet [11].

### Forschungsrichtungen

Ungeachtet der Einbeziehung des prozentualen GP4-Anteils in GS7 (GG2-3)-Pathologieberichten ermöglicht die Gesamtbetrachtung des relativen GP4- und 5‑Anteils eine bessere klinische Risikostratifizierung. Um kontinuierliche relative Anteile zu berücksichtigen, hat die Arbeitsgruppe aus der Martini-Klinik des Universitätsklinikums in Hamburg einen numerischen „integrierten quantitativen GS (IQ-Gleason)“ entwickelt, der von 0 (100 % GP3) bis 117,5 (100 % GP5) reicht. [12]. Der prognostische Wert dieses IQ-GS für das postoperative biochemische Wiederauftreten ist besser als das derzeitige Gleason-Grading und wurde bereits unabhängig validiert. [13].

Abgesehen von der besseren Trennschärfe hat die kontinuierliche Einstufung den zusätzlichen Vorteil, dass sie weniger anfällig für untersucherabhängige Variabilität ist. Liegt zum Beispiel eine PCa-Biopsieprobe mit 96 % GP3 und 4 % schlecht geformten Strängen mit spärlichen Lumina vor, bei denen man sich nicht sicher sein kann, ob man sie eher dem GP4 oder 5 zuordnen soll, würde die Zuweisung von GP4 zu GS3 + 4 = 7 (GG2) mit 4 % Muster 4 führen. Das würde bedeuten, dass dieser Patient für eine aktive Überwachung in Frage kommen könnte. Würde man hingegen zu dem Schluss kommen, dass das jeweilige Muster besser mit GP5 vereinbar ist, würde dies zu GS3 + 5 (GG4) führen, was als Hochrisikoerkrankung gilt und eine aktive Behandlung erfordert. Leider sind solche Extremszenarien nicht hypothetisch, sondern täglich erfahrbare Praxis. Die Anwendung einer kontinuierlichen Bewertungsstrategie wäre hier robuster, z. B. wäre der IQ-GS des ersten Szenarios 4 von 117,5 und des zweiten Szenarios 14/117,5, wodurch die Auswirkungen der willkürlichen Einstufungsvariabilität abgeschwächt würde. Ein weiterer neuerer Forschungsbereich bei der Quantifizierung ist die Frage, ob das absolute GP4-Volumen aussagekräftiger ist als sein relatives Verhältnis zu GP3. So hat beispielsweise eine GS4 + 3 = 7 (GG3) Biopsieprobe mit 2 mm PCa, die zu 70 % aus GP4 und zu 30 % aus Muster 3 besteht, eine absolute Länge von 1,4 mm für Muster 4. Eine GS3 + 4 = 7 (GG2)-Biopsie mit 12 mm PCa, die zu 80 % aus Muster 3 und zu 20 % aus Muster 4 besteht, hat dagegen 2,4 mm Muster 4.

Obwohl das absolute GP4-Volumen im zweiten Fall höher ist, ist kontraintuitiv das zugeordnete GS/GG niedriger. Dean et al. verglichen GP4-Quantifizierungsmethoden in GS3 + 4 = 7 (GG2)-Biopsien im Hinblick auf das Vorhandensein von ungünstigen Merkmalen bei der nachfolgenden RPE (definiert als ≥ GS4 + 3 = 7, ≥ pT3 und/oder Lymphknotenmetastasen) [14]. Sie fanden, dass der prozentuale Gesamtanteil an GP4, der maximale prozentuale Anteil an GP4 und die kumulative GP4-Länge alle signifikant mit „ungünstiger Pathologie“ assoziiert waren, dass aber die absolute GP4-Gesamtlänge die beste Aussage und den höchsten prognostischen Nutzen aufwies.

In einer neueren Studie kamen Vickers et al. [15] ebenfalls zu dem Schluss, dass die Quantifizierung des absoluten und nicht des prozentualen Anteils an GP4 am aussagekräftigsten für die Vorhersage einer ungünstiger Pathologie ist, und sie zeigten auch, dass das GP3-Volumen keinen signifikanten Einfluss auf dieses Ergebnis hat. Alle diese Daten befürworten die Quantifizierung des GP4 in unseren Befundberichten. Obwohl die klinische Bedeutung eher vom absoluten GP4-Volumen als von seinem relativen Verhältnis zu GP3 abhängen könnte, ist es derzeit nicht erforderlich, die absolute GP4-Länge anzugeben, da es zum einen nur wenige Vergleichsstudien gibt, zum anderen keine Standards für die einheitliche Berechnung und Angabe der GP4-Länge existieren und letztlich die Folgen für die klinische Entscheidungsfindung unklar sind.

## Das invasive kribriforme Muster

In den letzten 10 Jahren hat sich gezeigt, dass unter den GP4-Wachstumsmustern das Bestehen kribriformer Tumoranteile ein wichtiger und unabhängiger negativer prognostischer Parameter ist. So konnten Kweldam et al. [16] zeigen, dass Patienten deren Tumoren kribriforme Anteile in ihren Prostatabiopsien aufwiesen, signifikant verkürzte krankheitsspezifische Überlebenszeiten hatten. Das Vorhandensein von kribriformen Mustern wurde unabhängig voneinander mit fortgeschrittenem Erkrankungsstadium, postoperativem biochemischem Rezidiv, Versagen der Strahlentherapie, Lymphknotenmetastasen und Tod in Verbindung gebracht [17].

Aufgrund seiner prognostischen und klinischen Bedeutung wurde das kribriforme Muster vor kurzem detailliert definiert: Das kribriforme Muster ist gekennzeichnet durch eine konfluierende oder zusammenhängende Schicht maligner Epithelien mit mehreren interzellulären Drüsenlumina, die bei geringer Vergrößerung (10fache Vergrößerung) leicht zu erkennen sind; es sollte kein zwischenliegendes Stroma oder Muzin vorhanden sein, welches einzelne oder fusionierte Drüsenstrukturen trennt [18]. Folglich wurde auf der ISUP-Konsensuskonferenz 2019 und in der WHO-Klassifikation von 2022 festgelegt, dass das Vorhandensein eines kribriformen Musters immer im Befund vermerkt werden sollte. Dies ist auch für eine leitliniengerechte Befundung des PCa erforderlich (S3, LL 2025; [5, 6]).

### Forschungsrichtungen

Lediglich das Vorhandensein, nicht aber das Ausmaß des kribriformen Musters ist mit ungünstigen klinischen Verläufen verbunden, was bedeutet, dass selbst eine einzelne kribriforme Drüse ein Indikator einer aggressiven Erkrankung ist [16, 17]. Ob die Größe oder der Durchmesser eines kribriformen Drüsenfelds eine zusätzliche prognostische Bedeutung hat, bleibt zu untersuchen. Einige Studien belegten, dass Tumoren mit großen kribriformen Drüsen ungünstigere Verläufe zeigten, als Tumoren mit kleinen kribriformen Drüsen [19, 20].

Die Definition von großen kribriformen Mustern variierte jedoch von Studie zu Studie, diese umfassten: > 12 interzellulärer Lumina, eine größeren Ausdehnung als 0,25 mm bzw. mehr als die doppelte Größe der benachbarten gutartigen Drüsen [19–22]. So haben Greenland et al. [22] an einer Ektomiekohorte gezeigt, dass GG2-Patienten, deren Tumoren große kribriforme Anteile besaßen, häufiger ungünstige pathologische Parameter aufwiesen als Tumoren ohne bzw. mit lediglich kleinen kribriformen Drüsen. Andererseits stellten wir fest, dass das große kribriforme Muster in RPE-Präparaten mit ungünstigen histologischen Merkmalen und einem postoperativen biochemischen Rezidiven assoziiert war, dass allerdings sowohl ein kleines also auch ein großes kribriformes Muster bei der Biopsie einen ähnlichen unabhängigen prognostischen Wert für das metastasenfreie bzw. erkrankungsspezifische Überleben zeigten [20, 23].

Die Einbeziehung des kribriformen Musters in neuartige PCa-Graduierungsmodelle führt zu einer besseren Vorhersage der klinischen Verläufe als die derzeitige Gleason-Graduierung. So haben wir beispielsweise gezeigt, dass ein einfaches Grade-Modell, bei dem die GG um eins verringert wird, wenn kein kribriformes Muster vorhanden ist – mit Ausnahme des aktuellen GG1–, eine bessere Vorhersage des metastasenfreien Überlebens aufweist als konventionelle Graduierungsgruppen [24]. Darüber hinaus konnte vor kurzem eine Multicenterstudie zeigen, dass der Einschluss kribriformer Muster zu einer Verbesserung der NCCN- und der CAPRA-Risikostratifikationssysteme führt [25].

## Das intraduktale Karzinom

Das Konzept des intraduktalen Karzinoms (IDC) wurde 1986 durch McNeal eingeführt [26]. Sie definierten IDC als „vollständige Überbrückung des Duktus- oder Azinuslumens durch mehrere Trabekel bösartiger Epithelien, mit Herden trabekulärer Fusionen“. Sie stellten fest, dass die Prognose von Patienten mit IDC derjenigen von Patienten mit GP4 entspricht. 2006 legten Guo und Epstein [27] strenge morphologische Kriterien für die Diagnose von IDC bei Nadelbiopsien fest, diese umfassten eine intraduktale Proliferation maligner Epithelien mit einem der folgenden Muster: solide, dicht-kribriforme und/oder lockere kribriforme/mikropapilläre Muster mit entweder hochgradiger Kernatypie oder nichtfokaler Komedoknekrose. Zahlreiche Studien haben einen starken Zusammenhang zwischen dem Vorhandensein von IDC und hohem GS, großem Tumorvolumen, positiven chirurgischen Rändern, biochemischem Rezidiv und verkürztem krankheitsspezifischem Überleben gezeigt [16, 17, 27].

Obwohl es sich beim invasiven kribriformen GP4 und dem IDC um zwei streng voneinander getrennte pathologische Entitäten handelt, weisen sie erhebliche morphologische Überschneidungen auf und treten häufig zusammen auf [16]. Wie von mehreren Gruppen nachgewiesen, sind sowohl das Vorhandensein eines invasiven kribriformen Karzinoms als auch eines IDC in uni- und multivariablen Analysen signifikant mit einem schlechteren krankheitsspezifischen Überleben assoziiert [16, 17].

Aufgrund der morphologischen Überschneidungen und der ähnlichen Assoziation mit ungünstiger Prognose wurde auf der ISUP-Konsenskonferenz 2019 beschlossen, dass eine Unterscheidung zwischen beiden nicht erforderlich ist, wenn zugleich ein invasives Karzinom vorliegt. Dies bedeutet, dass Pathologen einfach das Vorhandensein eines „invasiven kribriformen und/oder intraduktalen Karzinoms“ diagnostizieren können. Eine weitere Schlussfolgerung ist, dass IDC, wenn es von einem invasiven Karzinom begleitet wird, in die Graduierung einbezogen werden kann. So kann beispielsweise eine Biopsie mit 90 % GP3 und 10 % IDC als GS3 + 4 = 7 (GG2) mit 10 % GP4 und Vorhandensein eines invasiven kribriformen und/oder intraduktalen Karzinoms angegeben werden. Im seltenen Fall von Biopsien, die nur IDC ohne invasives Karzinom enthalten, sollte das IDC nicht graduiert werden. Hier sollte allerdings kommentiert werden, dass der isolierte Nachweis eines IDC häufig ein Indikator höhergradiger, im Biosat aber nicht erfasster Tumoranteile ist. In diesen Fällen sollte eine sofortige Kontrollbiopsie empfohlen werden, auch eine umgehende Einleitung einer aktiven Therapie wird von einige Autoren erwogen.

## Atypische intraduktale Proliferation (AIP)

Die wichtigste Differenzialdiagnose des intraduktalen Karzinoms in der Biopsie ist die hochgradige PIN (HGPIN). Beide Entitäten weisen atypische zytologische Merkmale wie Kernvergrößerung, Hyperchromasie und vergrößerte Nukleoli auf. Während solide und dichte kribriforme Muster in dilatierten, präexistenten Drüsen keine architektonischen Merkmale einer HGPIN sind, ist die diagnostische Einordnung loser bzw. lockerer kribriformer Strukturen und nicht-expansiver kribriformer Muster in Drüsen oft schwierig. Solche mehrdeutigen Läsionen, die zwischen HGPIN und IDC liegen, werden als AIP bezeichnet [28, 29]. Shah et al. [28] stellten fest, dass das AIP in der Biopsie in den meisten Fällen ein Indikator für ein nicht erfasstes IDC ist und schlugen eine sofortige erneute Biopsie vor, um das Vorhandensein von höhergradiger Tumoranteile zu bestätigen. Da es sich bei der AIP nicht um eine nosologische Entität handelt, sondern eher um einen Ausdruck einer diagnostischen Unsicherheit, ähnlich atypischen kleinazinären Proliferaten (ASAP) für invasive Karzinomanteile, ist zu erwarten, dass konkretere Empfehlungen für die Diagnostik und die Klassifizierung der AIP noch zu etablieren sind.

## Aktuelle Entwicklungen

Aus einer Analyse der prognostischen Wertigkeit verschiedener morphologischer Muster heraus entwickelten McKenney et al. [30] ein Modell einer stark vereinfachten Graduierung, welche lediglich zwischen *ungünstigen* und *günstigen* Mustern unterscheidet. Unter ungünstigen Mustern fassten sie alle GP5-Anteile, große kribriforme Drüsen, IDC, stark stromogene Karzinome, komplexe intraluminale papilläre Architektur sowie Karzinome mit komplex-anastomosierendem Wachstum zusammen. Diese Signatur zeigt eine hochgradige prognostische Wertigkeit bezüglich des biochemischen Rezidivs, der Metastasierung und des Gesamtüberlebens, bedarf aber weiterer Validierung. Es ist derzeit nicht klar, ob diese Daten, die an RPE-Präparaten gewonnen wurden, ohne weiteres auf Stanzbiopsate übertragen werden können [31]. Schließlich birgt die klinische Anwendung künstlicher Intelligenz (KI) in der diagnostischen Prostatahistopathologie ein großes Potenzial. KI-basierte Algorithmen zur Gleason-Graduierung weisen eine hohe Übereinstimmung mit Pathologen auf, können die Variabilität zwischen den Beobachtern verringern und die Erkennung von kribriformen Wachstumsmustern unterstützen [32–34].

## Fazit für die Praxis

- Der Gleason-Score ist der Schlüssel zur Risikostratifizierung und klinischen Entscheidungsfindung bei Männern mit einem Prostatakarzinom.
- Die Integration der GP4-Quantifizierung in Pathologieberichte und Risikomodelle optimiert die Vorhersage und kann für die Beurteilung der Eignung zur aktiven Überwachung relevant sein.
- Das invasive kribriforme Muster, das intraduktale Karzinom (ICD) und das invasive duktale Karzinom sind sich morphologisch überschneidende Prozesse, die unabhängig voneinander mit schlechten krankheitsspezifischen Überlebenschancen verbunden sind.
- Obwohl es sich beim invasiven kribriformen GP4 und dem IDC um zwei streng voneinander getrennte pathologische Entitäten handelt, weisen sie erhebliche morphologische Überschneidungen auf und treten häufig zusammen auf.
- Die wichtigste Differenzialdiagnose des intraduktalen Karzinoms in der Biopsie ist die hochgradige PIN (HGPIN).
- Die künstliche Intelligenz birgt für die klinische Anwendung in der diagnostischen Prostatahistopathologie ein großes Potenzial.
